# Imagine the Superiority of Dry Powder Inhalers from Carrier Engineering

**DOI:** 10.1155/2018/5635010

**Published:** 2018-01-14

**Authors:** Piyush Mehta

**Affiliations:** Dry Powder Inhaler Lab, Respiratory Formulations, Cipla R&D, LBS Marg Road, Vikhroli (West), Mumbai, Maharashtra 400079, India

## Abstract

Inhalation therapy has strong history of more than 4000 years and it is well recognized around the globe within every culture. In early days, inhalation therapy was designed for treatment of local disorders such as asthma and other pulmonary diseases. Almost all inhalation products composed a simple formulation of a carrier, usually *α*-lactose monohydrate orderly mixed with micronized therapeutic agent. Most of these formulations lacked satisfactory pulmonary deposition and dispersion. Thus, various alternative carrier's molecules and powder processing techniques are increasingly investigated to achieve suitable aerodynamic performance. In view of this fact, more suitable and economic alternative carrier's molecules with advanced formulation strategies are discussed in the present review. Furthermore, major advances, challenges, and the future perspective are discussed.

## 1. Introduction

Delivery of therapeutic agents via pulmonary route has been studied meticulously in recent years. A huge surface area (80–120 m^2^), high permeability, easy transcytosis in alveolar region, extensive vascularization, and low enzymatic activity are prime factors for easy, successful, and efficient delivery of therapeutic agents into systemic and local area [1]. Therapeutic agents should have an aerodynamic diameter in range of 0.5–5.0 *μ*m to be inhaled and effectively deposited in the pulmonary area [1]. A variety of inhalation techniques have been developed and investigated to treat pulmonary disorders such as asthma, bronchiectasis, cystic fibrosis (CF), chronic obstructive pulmonary disease (COPD), pneumonia, and tuberculosis. Among them, dry powder inhalers (DPIs) are most commonly used techniques due to well recognized and quite evident benefits [2]. Few benefits of DPIs over other inhalation techniques are as follows: (i) no need for cold chain storage or reconstitution of powders, (ii) better physiochemical stability, (iii) satisfactory pulmonary deposition via the patient's respiration, (iv) easy incorporation of high mass of drug, and (v) superior opportunity of particle/carrier engineering [1, 2].

A DPI is a unique combination of micronized drug (1–5 *μ*m), suitable coarse carrier (90–150 *μ*m), and an appropriate inhaler device [2]. In dry powder inhaler system, carrier particles form an essential element because of small mass of drug inside the final formulation. The carrier presents bulk to the DPIs, which ease the dispensing, handling, and actuation of the micronized therapeutic agent, which is very essential for low dose DPIs, for example,* Spiriva® HandiHaler®* (*Boehringer Ingelheim*, typical dose: 18 mcg) [2]. Interaction among micronized drug and carrier molecules has gained a lot of consideration in scientific literature. Numerous processing techniques deliver the cohesive drug powder with high surface energy, whereas addition of the carrier controls the cohesiveness of the micronized drug and enhances aerosolizing [3]. Micronized drug particles should freely adhere to the carrier molecules and at time of inhalation drug particles should easily separate from the carrier particles and are made available for dispersion into the lungs [2]. The adhesion force between carrier and micronized drug particles is mainly governed by three forces: (i) molecular force (van der Waals forces); (ii) coulombic force (electrostatic charges); and (iii) capillary force (moisture content) [4]. Among the forces, molecular forces are most important forces from the viewpoint of adhesion or cohesion in final formulation and dispersion/deagglomeration during the inhalation [4]. Adhesion forces and contact area between micronized drug and carrier particles are also influenced by carrier shape and surface morphology, respectively [5]. Equilibrium between adhesive-cohesive forces in the powder mixture and separation effort produced by the deagglomeration principal during inhalation should be adjusted to confirm the maximum powder homogeneity with reliable content uniformity and reproducible fine particle fraction (FPF). Until now, variables, mechanisms, and various mixing theories involved in the drug-carrier and drug-drug interaction are also judged and analyzed by numerous formulation scientists. The considerable attention has also focused on the presence and amount of carrier (lactose) fines in final formulation. Various hypotheses related to “addition of fines” are summarized in Table 1 [6, 7]. Hence, a small change in carrier physicochemical properties such as carrier particle shape, size, density, surface area, surface energy, surface roughness, electrostatic charge, surface morphology, and topography have a substantial effect on drug aerodynamic performance [8]. *α*-LM is the most regularly and recurrently used carrier in various commercial DPI formulations. Existing lactose based DPI formulations from different manufacturers have been revealed to differ in the particle size distribution (PSD), amorphous content, water content, and presence of fine and surface characteristic, all of which may cause altering drug aerosolization behavior (Table 2) [9, 10]. Thus, most appealing tool to augment the efficiency and effectiveness of dry powder inhaler formulations is utilizing of engineered carrier particles or therapeutic agents. Excipients suitable for use in DPIs-based pulmonary delivery are listed in Table 3.

Particle engineering is a novel process that unites fundamentals of biomaterials, colloid and interface science, nanotechnology, solid state physics, materials chemistry, and formulation science [11]. The recognition and popularity of particle engineering technique can be realized by the fact that many academic and industrial research institutes and universities globally are analyzing and investigating different aspects of this particle engineering technology [12]. PubMed presents huge scientific publications (34962) from 1950 to till date when searching for the phrase “particle engineering.” Approximately 2% of these scientific publications were located under “particle engineering inhalation,” therefore demonstrating vast curiosity of researchers in particle engineering as a capable approach for pulmonary drug delivery. Practicability of particle engineering for DPI carrier engineering has been comprehensively discussed in several peer review research articles. During the last few years, there has been a rising interest in the use of modified carriers as drug carriers [13]. There are numerous particle engineering techniques available to synthesized inhalable carrier particles. Different particle engineering techniques summarize the final characteristics of carrier particles. Selection of an appropriate technique depended on the criteria of particle shape, size, pleasing surface properties, and morphology of carrier molecule [11, 12]. Numerous conventional and newly developed techniques are available to synthesized inhalable carrier particles such as micronization and blending, spray-drying (SD), spray-freeze drying (SFD), freeze drying (FD), supercritical fluid (SCF), supercritical antisolvent (SAS), ultrasound assisted antisolvent (UAS), and most advance techniques like high-volume production methods, that is, electrospinning [11, 14]. The development of carrier engineering based DPIs has the capability to defeat problems linked with carrier as a vital constituent of the formulation [15]. Various therapeutic agents, such as proteins, peptides, antibiotics, anticancer drugs, and steroids, can be easily loaded on engineering carrier particles for improving* in vitro* and* in vivo* aerosolization pattern [16].

Numerous promising DPIs have been effectively delivered using carrier engineering technique. The first section of this review presents the different engineered sugars, polyols, and dextrin's molecules that have been used for the various DPI formulation, with a special emphasis on manufacturing techniques resulting in versatile carriers with controlled drug release properties. A brief account of each sugars, polyols, and dextrin's molecule is prefaced for those readers not familiar with the molecules. The second section of the manuscript is dedicated to dry powder coating technique and force controlling agents including their influence on drug dispersion and disposition pattern. This manuscript abridges the advancement that has been made in the last few years in the field of carrier engineering and powder processing techniques for effective DPI delivery and highlights the key challenges that remain to be answered.

## 2. Sugars and Polyols Carriers for DPI

### 2.1. Lactose Monohydrate

**Figure 1:**
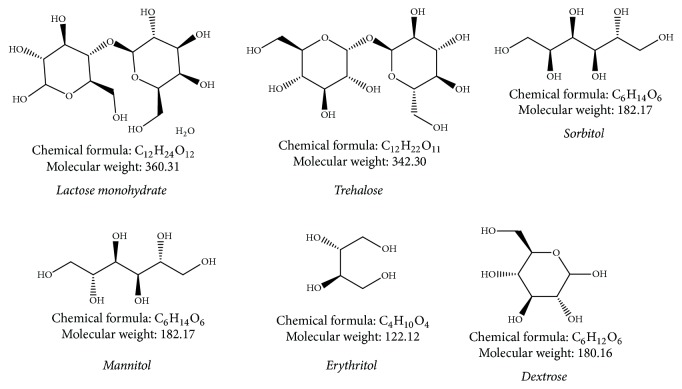
Chemical structure of various carriers used in DPI formulations.

Lactose is a carbohydrate and as such a disaccharide. It is composed of galactose and glucose, linked by a *β*-l-4 glycosidic bond. The molecular constitution of lactose is showed in Figure 1. In milk, lactose is habitually found as either *α*-lactose or *β*-lactose or amorphous glass form (mixture of *α*- and *β*-forms). Molecular structures of *α*- and *β*-lactose vary in the orientation of a hydroxyl group and a hydrogen at C_1_ in the glucose moiety [17]. Both forms transform into each other persistently. This incident is termed mutarotation. Rate of mutarotation is governed by factors like concentration, temperature, and pH (acidity) of solution. At normal room temperature stability is achieved in a ratio of almost 40%  *α*-lactose and 60%  *β*-lactose. Two different forms of lactose have great consequence on a variety of physicochemical characteristics of lactose such as crystal morphology, crystallization behavior, solid state properties, and solubility. Lactose in solid form can also be in an amorphous state or a crystalline state. Apart from *α*- and *β*-lactose, two crystalline anhydrous *α*-lactose types are recognized, a stable and an unstable (hygroscopic) form. Moreover, a so-called mixed crystalline form is noted, enclosing both *α*- and *β*-lactose in a unique crystal lattice [18].

#### 2.1.1. Crystallized Lactose Particles

Augmenting effectiveness of drug aerosolization even now offers a key dispute in progress of DPI formulations. A small alteration in carrier physicochemical characteristic is prone to have a significant consequence on drug* in vitro* aerosolization pattern. Therefore, use of appropriate engineered carrier molecule could be a crucial aspect for enhancing DPI functioning [19]. Some approaches have been studied to complete this assumption together with batch cooling crystallization [20], antisolvent crystallization [8], and so on. These techniques were utilized to alter lactose carrier particles, to enhance aerosolization of drug from DPI formulations. Antisolvent crystallization, also called salting out, drowning out, or precipitation crystallization, is used to generate a solid from a solution in which the product has high solubility. This technique is commonly used for polymorph control, purification, and yield improvement. Antisolvent crystallization accomplishes supersaturation and solidification by exposing a solution of the substance to another solvent (single or multiple) in which the substance is sparingly soluble [21]. It has been studied that characteristics of an antisolvent crystallized substance are reliant on some processing factors, for example, solution concentration, type of antisolvent, antisolvent addition rate, agitation intensity, and mixing conditions [22]. Antisolvent crystallization methods with various binary nonsolvents including acetone-water [23], ethanol-butanol [24], ethanol-acetone [25], and acetone-butanol [26] formed lactose particles which when integrated into DPI formulations of salbutamol sulphate (SS) led to an improved aerosolization performance. In comparison to commercial lactose (CL), engineered lactose particles showed variable elongation ratio and shape. Additionally, the CL was in *α*-LM form, but engineered lactose particles were mixtures of *α*- and *β*-lactose [25]. Crisp et al. showed that crystal size improved as antisolvent quantity diminished [27]. Antisolvent crystallization using alcohols does suffer from two major disadvantages, that is, solvent recouping and hazard coupled to use of flammable solvents [28].  Table 4 summarizes some appealing research on antisolvent engineered carrier particles integrated DPI formulations. Continuous mechanical stirring during crystallization can set off arbitrary energy fluctuations inside solution beginning an assorted distribution of local concentrations, leading to heterogeneous crystal development. Alternatively, particles with a uniform particle shape and a narrow size distribution can be formulated by suspending the crystals in a gel, where secondary nucleation (heterogeneous nucleation) happens to a much lesser extent [20]. Zeng et al. prepared inhalable crystalline lactose particles using Carbopol 934 gels (carbo lactose). Developed elongated smooth surface crystals showed tomahawk shape with narrower PSD and better flowability compared to *α*-LM of a similar particle size [29]. Same research group [30] also prepared SS-loaded DPI formulation using carbo lactose for pulmonary delivery. Smooth surface carbo lactose showed a narrow size distribution and better flowability as compared with commercial grade lactose (Lactochem®). Aerodynamic dispersion of SS was studied using a MSLI with Rotahaler® device using hard gelatin capsules. Liquid impinger analysis suggested good dispersion and deposition with FPF of 21.50 ± 1.2%. Crystalline lactose particles showed ~1.50-fold improvement in % FPF compared to commercial grade lactose [29, 30]. Recently, Patil et al. research group developed respirable crystalline lactose particles by liquid crystalline phase technique for pulmonary delivery of SS. Developed elongated lactose crystals showed cubic mesophase with smooth surface, 1.56 ± 0.06 elongation ratio, 71.66 ± 6.27 *μ*m particle size, and good flowability. Aerosol dispersion was examined by a ACI with Rotahaler device at flow rate of 28.3 l/min using hard gelatin capsules. ACI analysis suggested good dispersion and deposition with FPF, ED (emitted dose), and RD (recovered dose) of 47.44 ± 1.62%, 363.37 ± 9.99 *μ*g, and 447 ± 27.16 *μ*g, respectively. Results showed that liquid crystalline phase-crystalline lactose DPI can be effective delivery option to achieve a high local concentration [31]. Dhumal et al. developed and analyzed inhalable formulation composed of ultrasound assisted lactose crystals as an effective pulmonary carrier for SS. The developed lactose crystals showed elongated rod shape with narrow PSD (~79 *μ*m) and better flowability.* In vitro* testing was studied using a ACI with Rotahaler at flow rate of 28.3 l/min by size #3 hard gelatin capsules. ACI analysis showed good dispersion and deposition pattern with the FPF, ED, and RD of 27.82 ± 1.5%, 381 ± 12 *μ*g, and 95.63 ± 1.3%, respectively. Prepared lactose crystals showed ~2.15-fold improvement in aerodynamic performance compared to Pharmatose® 200 M formulations. The improvement in deposition profiles may be accredited to the variation in the morphological properties like high elongation ratio and surface smoothness [32]. Microspherical lactose crystals were prepared by the supercritical carbon dioxide (scCO_2_) fluid approach in the presence of a methanol to improve crystal morphology. Lactose crystals formed by scCO_2_ method showed satisfactory FPF (40 ± 0.3%) for SS when determined using the Aerolizer® at a flow rate of 28.3 l/min. This was considerably superior to that of commercial coarse grade lactose which was found to be 38% [33].

#### 2.1.2. Spray-Dried Lactose

Spray-drying (SD) is a unit operation that transfers a fluid supply to a dried particulate. The feed can be simply a solution, a colloidal dispersion (e.g., emulsion, liposome), or a coarse or fine suspension, which is initially atomized to a spray form that is placed instantly into thermal contact by means of a hot air, leading to quick evaporation of droplets to materialize dried solid particulates. The dried particulates are then isolated from the gas by way of an electrostatic precipitator, cyclone, or bag filter. Key rationale of aerosolizing spray-dried powders is to attain desired PSD. This guarantees a suitable aerodynamic performance with a satisfactory pulmonary deposition of drug in deep alveoli areas at regular inhalation rates. The key advantage of SD regarding pulmonary delivery is capability to control and direct a range of factors, for instance, solute concentration, solution feed rate, solvent composition, relative humidity, and temperature. This permits optimization of particle features such as PSD, shape, density, and surface morphology. SD is a frequently employed system in pharmaceutical R&D for developing dry powders for inhalation carrying various drugs, for instance, beclomethasone, budesonide, SS, or peptides and proteins such as deoxyribonuclease and insulin [34].  Table 5 illustrates various investigations on the SD based carrier particles for better functionalities of inhaled drug molecules.

#### 2.1.3. Electrohydrodynamics Assisted Lactose Crystals

Electrohydrodynamics atomization is the electrostatic interactions based technique used for continuous production of long solid, nonwoven, hollow, ribbon-shaped, and nanoporous nano/microfibers with variety of natural and synthetic polymers and various composites [39]. These matrices have recently become more attractive platform for drug delivery applications because of their unique mechanical and physicochemical properties like large surface area, high loading capacity, good porosity, and concurrent delivery of diverse molecules. This versatile technique is also effectively used for production of inhalable particles with controlled sizes and shapes [39].

Very recently, lactose, which is a normally used excipient in pulmonary drug delivery, was electrosprayed by E-Spin Nanoelectrospinning apparatus. The obtained outcomes recommended that the electrospraying process produced elongated and smooth surface lactose crystals with *α*- and *β*-isomer mixture (<80 *μ*m). Aerodynamic performance was determined using TSI with Rotahaler device at flow rate of 60 l/min for 4 secs using #3 gelatin capsules. TSI analysis presented good dispersion pattern for electrospray lactose with FPF and ED of 18.15 and 74.52% for SS, respectively. Therefore, to conclude, electrospray lactose particles may be successfully used for delivery of various pulmonary therapeutic agents [40].

#### 2.1.4. Miscellaneous Lactose Carriers

Irregular-shaped fragmented (<100 *μ*m) lactose particles with rough surface (*Ra*; 1355 nm) have been produced by roller compaction (RC) a feed containing Spherolac® 100 and Granulac® 200 (1 : 1) for pulmonary delivery of isoniazid. Developed lactose particles possessed FPF of >35% and showed surprisingly good aerosolization pattern when measured via a Rotahaler device at 100 l/min [41]. Young et al. prepared modified lactose surfaces using proprietary process (i.e., particle smoothing) for pulmonary delivery of beclomethasone dipropionate (BDP). Developed lactose particles showed 178.8 ± 0.4 *μ*m particle size (*d*_90_), 12.2 ± 2.9 nm *Ra*, and 32.00 *μ*J separation energy (*e*_0.9_) with a smooth surface structure. Developed lactose particles showed 4.21-fold improvement in fine particle dose (FPD) as compared to untreated lactose when measured via a Multidose Inhaler device at 60 l/min. This particle processing technique could thus helpfully be used to improve aerodynamic performance of DPI formulations and as a substitute to the current processing techniques [42].

Various approaches have been available regarding fabrication and characterization of *α*-LM for inhalation purposes. However, because of clinical concerns, *α*-LM cannot be exercised for drug delivery to diabetic patients. Furthermore, for some molecules (e.g., formoterol) or for specific purpose (e.g., protein or peptide molecules) *α*-LM possibly will not be the carrier of preference as an outcome of its reducing sugar functionality that can interact with functional groups of drug molecules [43]. In addition, *α*-LM is obtained from bovine milk or with bovine-driven additives so that transmissible spongiform encephalopathy is even now a problem of worry for this molecule. Lactose intolerance is a trouble that demands patient to use lactose-free formulations. It is thus rational to search for alternative carriers that still own the optimistic features but defeat the above stated shortcoming of *α*-LM [5].

### 2.2. Mannitol

Mannitol is d-mannitol (Figure 1). It is a hexahydric alcohol associated with mannose and is isomeric with sorbitol. It is a nonreducing sugar, is less hygroscopic than lactose, and has a sweet taste roughly as sweet as glucose and half as sweet as sucrose and offers a cooling sensation in mouth. It occurs as a white, odorless, crystalline powder or free flowing granules. It has been largely used as a pharmaceutical excipient. It is also effectively used as a carrier for pulmonary delivery. It is confirmed to be most proficient excipient for this application compared with more hygroscopic sugar alcohols such as maltitol, sorbitol, and xylitol [5, 44].

#### 2.2.1. Crystallized Mannitol Particles

The morphology and drug dispersion of crystallized mannitol particles (CMP) were mainly reliant on the proportion water in nonsolvent and crystallization technique employed [19]. Antisolvent crystallization techniques using multisolvent including acetone-water [45], ethanol-water [46], and ethanol-acetone [47] formed CMP are incorporated into DPI formulations of SS. Developed formulation displayed better aerosol performance than commercial mannitol (CM), despite the ratio of antisolvent utilized in the recrystallization procedure. An adjustment in ratio of ethanol-water or acetone-water heads towards mannitol with a different polymorphic content [45, 46].* In vitro* aerodynamic analysis showed better dispersion and deposition pattern for CPM obtained from lower supersaturation 20% (w/v) (% FPF, 31.6 ± 2.3%) compared to CPM with high supersaturation 50% (w/v) (% FPM 14.2 ± 4.4%). Better aerodynamic behavior of CPM obtained from lower supersaturation degree was credited to smoother surface, high elongation ratio, and higher “intrinsic” fines content of developed formulation [19]. SS DPI was formulated by mixing of lactose carrier with different crystallized mannitol particles [e.g., cooling crystallized mannitol (CCM), acetone-crystallized mannitol (ACM), and ethanol-crystallized mannitol (ECM)]. Aerosol dispersion was examined by a MSLI with Aerolizer device at flow rate of 92 l/min using size #3 hard gelatin capsules. The ternary formulation containing ECM particles demonstrated satisfactory aerosol functioning of FPF, 32.6 ± 1.1% with 2.18-, 1.14-, and 1.13-fold improvement in FPF compared to lactose alone, CCM, and ACM, respectively. Improvement in aerosol functioning of ECM may be credited to elongated particles with weaker particle adhesion force [48]. Kaialy and Nokhodchi developed mannitol carriers by treating CM by means of supersaturated 25% (w/v) mannitol aqueous solutions for 24 hr. Developed smooth surface microparticles were composed of *β*-mannitol with *Ra* of 1.2 ± 0.8 nm. Aerosol dispersion was examined by a MSLI with Aerolizer device at flow rate of 92 l/min by size #3 hard gelatin capsules. Prepared mannitol carriers showed satisfactory FPF of 35.9 ± 1.1%. The outcomes recommended that mannitol particle surface texture controls both particle size and shape of mannitol in terms of shaping* in vitro* functioning of mannitol [49]. Various investigations on crystallized mannitol particles are listed in Table 6.

#### 2.2.2. Spray-Dried Mannitol Carriers

SD was utilized to fabricate variety of mannitol particles varying in size, shape, and *Ra*. Littringer et al. formulated mannitol carrier particles of customized *Ra* using aqueous mannitol (15%, m/m) solution by SD technique. At lower outlet temperature, that is, 67°C (M67), carrier surface is composed of rod shaped crystals (122.4 ± 0.8 *μ*m) with mean *Ra* value of 140.33 ± 27.75 nm. When the powders were evaluated using a Novolizer® device at flow rate of 78.2 l/min, the highest FPF was obtained in a M67 carrier formulation (~30%). Mean *Ra* and particle shape showed significant impact on uncoupling of drug particles from carrier. Highest % FPF was obtained by carrier particles of rough surface and a spherical shape [51]. DPI formulations of composite mannitol were developed by Young and coworkers using a SD technique. Developed composite carriers are composed of lots of minor subunits rather than one crystal plane with 1.95 ± 0.63 elongation ratio, 2.72 *μ*m VMD (*d*_0.5_), and 0.79 ± 0.09 *μ*m *Ra*. Delivery of these composite particles via Cyclohaler® device at a flow rate of 60 l/min resulted in a calculated FPF of ~19%, a statistically considerable enhancement in FPF compared to the CM carrier (~6%) [52]. Kaialy et al. fabricated albuterol sulphate DPI formulation, using spray-dried mannitol (SDM) particles and compared these particles to the commercial mannitol and lactose carriers. The SDM particles were uniform and spherical with homogeneous PSD but composed mixture of *α*- and *β*-mannitol crystal with absence of *δ*-mannitol. A satisfactory FPF (>25%) was seen for the formulation containing SDM carriers. SDM carriers, which showed satisfactory* in vitro* deposition pattern, exhibited 1.73- and 1.30-fold increase in* in vitro* MSLI respirable fraction and superior dose uniformity compared with the commercial mannitol and lactose, respectively [53]. Tang et al. developed and studied aerosolization behavior of confined liquid impinging jets (CLIJs) followed by jet milling process mannitol powders. CLIJ routed mannitol exhibited a lower FPF (~30%) compared with the SDM (~46%), but CLIJ route presented some other essential compensation. Major advantage of CLIJ method is a satisfactory yield and better collection capability compared to SD technique [54].

#### 2.2.3. Freeze Dried Mannitol

However, SD process has some disadvantages, for example, reduced crystallinity for SD products, being not appropriate for material prone to mechanical shear (e.g., proteins, peptides, and hormones), and being not appropriate for materials that are unsteady for liquid-air interface or decayed by oxidation and poor process yield [55]. Freeze drying (FD) is a system by which it is feasible to improve process yield from aqueous solutions. Thus, compared with SD, FD displays more advantages in process yield, stability, and mechanical shear. Freeze dried mannitol (FDM) particles were prepared by using 5% (w/v) aqueous mannitol solution. The resultant mass was passed through a mechanical shaker to obtain particles with uniform size fraction, that is, 63–90 *μ*m. The FDM particles were elongated, porous, and irregular-shaped but composed mixture of *α*-, *β*-, and *δ*-mannitol. Aerodynamic delivery of the engineered FDM using Aerolizer device at a flow rate of 92 l/min resulted in a calculated FPF of 46.9%, a statistically substantial augmentation in FPF compared to the CM (16.8%) and SDM (24%) formulation. The superior aerosolization performance of the irregular-shaped particles was justified by their narrow PSD, moderate adhesive forces, and good flow properties [55]. Aerodynamic performance of different sieve fractions was further tested by Kaialy and Nokhodchi, who confirmed that large size fractions (90–125 *μ*m) were more useful than smaller particles since they formed DPI formulations with better drug content and flowability with satisfactory drug depositing in lower airways. Inhalable fractions (obtained with a MSLI at 92 l/min) ranged between 37.5 and 48.6% for all FDM sieve fractions formulations, while that for the CM and CL was only approximately 17 and 20% [56]. Kaialy and Nokhodchi [57] developed and studied the aerodynamic and physicochemical characteristics of a leucine-containing FDM for the effective delivery of hydrophobic steroid, that is, budesonide. PSD of the engineered powders was narrow and monomodal with >90% of the particles having a diameter around 300 *μ*m. Aerodynamic delivery of developed leucine-FDM using Aerolizer device at a flow rate of 92 l/min resulted in a better FPF of 35%, due to high porosity and elongated shaped carrier particles. Leucine coating also resulted in a 4.6- and 1.2-fold improvement in FPF compared to the CL and FDM alone formulation [57]. In addition to above-mentioned examples, various investigations on composites mannitol carrier particles are listed in Table 7.

### 2.3. Trehalose

Trehalose dihydrate (Figure 1) is a disaccharide and crystalline hydrate like lactose. But, it is a nonreducing sugar and *α*-LM is a reducing sugar [44]. *α*,*α*-Trehalose is only naturally occurring isomer of trehalose and appears as dihydrate. However, *β*,*β*-trehalose (iso-trehalose) and *α*,*β*-trehalose (neotrehalose) have been produced and are also accessible commercially. Trehalose is obtained as virtually odorless, white, or almost white crystals with a sweet taste (~45% of sweetness of sucrose) [62]. It is a nonreducing sugar and thus does not react with small molecular weight drugs (e.g., formoterol and budesonide), amino acids, or polypeptide- or protein-type drugs as a part of Maillard browning. It is moderately stable below low-pH circumstances calculated for other disaccharides. Hence, some efforts have been exerted on application of trehalose in DPIs [5, 62].

#### 2.3.1. Engineered Trehalose Particles

Trehalose dihydrate as an aerosol drug-carrier for DPI delivery has been systematically studied and was found to be an effective alternative to *α*-LM [63]. It has been proved that trehalose can efficiently improve dry powder aerosolization by increasing the FPF [64]. FD and SD are the two most general processes which allow the transformation of the formulation from the liquid state to the solid state mainly for DPI formulations [65]. However, freezing and thermal stresses related to both processes are frequently observed. Thus, cryoprotectants and thermos-protectants (e.g., carbohydrates) are regularly included in the formulation for the reason of protection of drug stability [66]. Trehalose can be effectively incorporated in inhalation formulations that contain biotherapeutic drugs (biologics), including aqueous solution spray-freeze-dried formulations to construct large trehalose particles for nasal vaccination delivery [67]. Trehalose has been used a lyoprotectant stabilizing excipient in DPI formulations [68]. It has many advantages over other sugars, such as less hygroscopicity, low chemical reactivity, a lack of inner hydrogen bonds that permits more adaptable formation of hydrogen bonds with drugs, and high *T*_*g*_ [69]. Additionally, trehalose dihydrate has distinguished similarities to the pulmonary sugar carrier, lactose monohydrate, as both are crystalline disaccharides and both are hydrates. Trehalose dihydrate is a nonreducing sugar, and, hence, it is not sensitive to the* Maillard reaction* (a solid state chemical degradation process). Moreover, it is not metabolized by bacteria due to its nonreducing property [66]. Pulmonary immunization has newly achieved improved awareness to persuade equally mucosal and systemic immunity while reducing problems linked to use of needles in parenteral vaccination. However, in comparison to respiratory administration of small molecule drugs, carrier engineering platform is easily adjustable to integration of biomacromolecules (e.g., hormones, vaccine antigens, proteins, and peptides) as a universal measure is lacking [2]. Sou et al. studied aerosolization pattern of spray-dried trehalose-leucine (9 : 1) loaded influenza vaccine powders. The physicochemical characteristics of developed microparticles are glass transition temperature (*T*_*g*_) 57.3°C and water content approximately 4%. Aerosol dispersion of these designed microparticles using Monodose Inhaler device at a flow rate of 90 l/min resulted in FPF of approximately 50%. Furthermore, developed formulation was not toxic to human lung tissues and resulted in better systemic and mucosal immunity [70]. Inhalable trehalose microparticulate and/or nanoparticulate powders were successfully formulated by SD technique. Developed microparticulate/nanoparticulate consists of amorphous trehalose with low amount of water content. Although no* in vivo* indication was investigated for developed trehalose particles, this system might be functional in pulmonary delivery of various drug molecules [66]. Ógáin et al. merged the delivery and drug release capability of nanoporous/nanoparticulate microparticles (NPMPs) systems with the simplicity of processing and aerosolization capability of microparticles by SD hydrophilic material, trehalose and lysozyme (4 : 1). Developed amorphous NPMPs showed particle size of 2.48 ± 0.04 *μ*m and *T*_*g*_ of 124°C. The NPMPs presented MMAD of 2.7 *μ*m and FPF of ~40% and might be an effective option for pulmonary delivery of protein/peptide molecule [71]. Generally, trehalose exhibits *T*_*g*_ of 117°C, based on existence of water and other excipients, likely to form amorphous matrix after SD, and has been utilized to stabilize proteins/peptides during storage in some investigations. Even at high concentration trehalose forms extremely adhesive, hygroscopic amorphous matrix which leads to high degree of particle agglomeration and reduces aerodynamic performance [72]. Therefore, multicomponent carrier mixture can be an option to augment aerodynamic performance and solid state characteristic of trehalose containing DPIs. Trehalose-leucine (80 : 20) microparticles of SS were fabricated by the co-SD technique. Aerosolization of microparticles via a Monodose Inhaler device led to a FPF of more than 65%. FPF values of the spray-dried microparticles differ, depending on carrier molecule and leucine content, ~81% (mannitol-leucine; 90 : 10) and ~67% (trehalose-leucine; 80 : 20) [72]. Superior* in vitro* aerodynamic outcomes were noted for mixture of albuterol and ipratropium with trehalose dihydrate in contrast with *α*-LM and mannitol [73].

### 2.4. Erythritol

Erythritol, a mesocompound of 1,2,3,4-butanetetrol (Figure 1), is a naturally occurring noncalorific polyol existing in several fruits and fermented foods, as well as in body fluids of humans and animals [44]. It is occurring as a white or almost white powder or granular or crystalline substance and industrially it is prepared from glucose fermentation. It has pleasant taste with a mild sweetness ~60–70% that of sucrose. It also has a high negative heat of solution that provides a strong cooling effect [62]. It has been administered as an apt excipient in pharmaceutical preparations because of its very low hygroscopicity, thermal stability, sweet taste, and low toxicity [44]. Various investigations on erythritol carrier based formulations of inhaled drugs are discussed below.

#### 2.4.1. Erythritol Microparticles

Glucagon is a 29-amino-acid peptide and essential element of glycogen metabolism and is known to be useful in clinical therapy of hypoglycemia and control of normal glucose levels in patients suffering from pancreatectomy, though medical application of this gut hormone has been limited to parenteral delivery. Pulmonary delivery of these proteins and peptides could be considered as an effective alternative route to parenteral delivery. Endo et al. reported an attractive study of glucagon/erythritol (1 : 100) microparticles developed by using conventional milling approach. Aerosol dispersion of these microparticles using Jethaler® device at a flow rate of 28.3 l/min resulted in FPF of approximately 34%.* In vivo* transpulmonary pharmacokinetic studies in rats exhibited 2.5% improvement in blood glucagon level compared to glucagon injection which can be justified by the higher FPF of microparticles [74]. The objective of the investigation performed by Traini et al. was to compare aerodynamic behavior of erythritol and *α*-LM for pulmonary delivery of SS. FPD values showed that *α*-LM was ~2-fold more effective than erythritol carrier. Although erythritol demonstrates a lower performance, it may present some probable advantages in terms of its reproducible chemical structure and stability [75].

### 2.5. Sorbitol

d-Sorbitol, less commonly known as d-glucitol, is a polyhydric alcohol with about half the sweetness of sucrose (Figure 1). Sorbitol occurs naturally from many edible fruits and berries and is also produced synthetically from glucose and corn syrup [44, 62]. It is related to mannose and is an isomer of mannitol; they differ only in orientation of the hydroxyl group on carbon number 2. Sorbitol appears as an odorless, white or almost colourless, crystalline, hygroscopic powder. It has been largely used as a pharmaceutical excipient. It is also effectively used as a carrier for aerosol delivery [62].

#### 2.5.1. Sorbitol Carrier Particles

Reducing sugars, for instance, *α*-LM, can have an influence on stability of amino acids or polypeptide- or protein-type drugs. In fact, use of *α*-LM with polypeptide- or protein-type drugs may lead to a reaction with lysine residues subsisting in protein and produce lactosylated protein molecules [44]. In this situation, polyhydric alcohol, for instance, sorbitol, may perform a key role in development of inhalable protein powder. Sorbitol may also act as a stability enhancer during development. Stability of interferon-*β* respirable powder was based on total sorbitol content of formulation [44]. Respirable powder aerosolization for SS was determined with binary and tertiary mixture of lactose, mannitol, and sorbitol as coarse and fine carriers. The dispersion and deaggregation of SS were examined by a TSI with Rotahaler device at flow rate of 60 l/min using size #3 hard gelatin capsules (Capsugel®, Belgium). Sorbitol demonstrated almost similar aerodynamic behavior for SS with lactose and mannitol but is unable to generate effective dispersion of drug (FPF < 10%) [76].

## 3. Functional Dextrin's Molecules for DPI Formulations

### 3.1. Cyclodextrin

**Figure 2:**
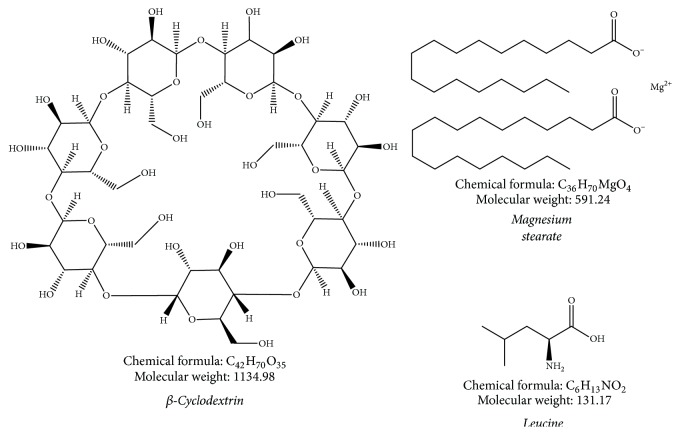
Chemical structures of various carriers used in DPI formulations.

Cyclodextrins (CDs) are cyclic oligosaccharides derived from starch with hydrophobic central cavities and hydrophilic outer surfaces. They create inclusion complexes with drugs, where drug links noncovalently to CD by partial or complete insertion into hydrophobic space. There are 3 forms of CDs used in pharmaceutical formulations: they are *α*-, *β*-, and *γ*-CD (Figure 2). The classification is depending on amount of glucopyranose units in a CD ring; *α*-, *β*-, and *γ*-CD have six, seven, and eight units, correspondingly [77]. The means by which CDs confer controlled release of a drug can vary. One assumption is that CDs delay drug release through dissolution-limited kinetics. CDs can be utilized to augment drug solubility and dissolution rate, revise release kinetics, increase stability, control local irritation, and decrease volatility of drugs. CDs maybe act as a flexible platform for pulmonary drug delivery systems [78]. Therefore, next segment summarizes applications of CDs alone and with other excipients.

#### 3.1.1. *β*-CD Microparticles Carrier

Trehalose/2-hydroxypropyl-*β*-cyclodextrin (HP-*β*-CD) (0.04% m/m, lyoprotectant) microparticles carrier was formulated using spray-freeze drying technique to investigate the feasibility of incorporating a linear polypeptide hormone, that is, salmon calcitonin, into trehalose/HP-*β*-CD carrier system. Developed spherical porous microparticles carrier showed rough surface with particle size of 26.17 ± 0.06 *μ*m. Dispersion of microparticles using a Cyclohaler led to a FPF of ~54% and ED of >45%. FPF values of developed microparticles showed that trehalose/HP-*β*-CD were about 1-fold more effective than a maltose/HP-*β*-CD system. Additionally, developed microparticles carrier showed satisfactory aerosol performance during three-month stability testing [77]. NPMPs of raffinose pentahydrate and trehalose dihydrate were fabricated by typical co-SD approach in presence of a cyclic oligosaccharide (HP-*β*-CD) to enhance its dispersion. NPMPs produced by co-SD the raffinose/HP-*β*-CD (40 : 60) and trehalose/HP-*β*-CD (30 : 70) and exhibited a FPF of >85% when concluded via HandiHaler using size #3 hard gelatin capsules. Moreover, developed NPMPs showed satisfactory physicochemical properties and physical stability [79].

#### 3.1.2. Highly Branched Cyclic Dextrin

Highly branched cyclic dextrin (HBCD) is an innovative form of dextrin that is obtained from waxy corn starch by cyclization reaction of a branching enzyme transferase. HBCD has been believed to be a type of hydrodynamic aggregation cluster with a diameter of several tens of nanometers in aqueous solution, as in many glycosylated food additives [80]. A branching enzyme is involved in formation of branch linkages (*α*-1,6 linkages) of starch and glycogen, and expected structure of HBCD consists of either a cyclic cluster or helical structure. This dextrin is very soluble in aqueous medium and has a moderately poor propensity for retrogradation. HBCD might improve the solubility or absorption of drugs by composite structure. Thus, HBCD could embed drug in aggregated structures irrespective of hydrophobic and hydrophilic nature [81]. The possibility of HBCD as an excipient matrix in DPIs was tested.

#### 3.1.3. HBCD Decorated Microparticles

Kadota et al. developed rifampicin/HBCD (1 : 10) loaded microparticles via SD method and studied the aerodynamic and deposition pattern using a Jethaler at flow rates of 28.3 l/min. Developed wrinkled shape porous microparticles consist of amorphous rifampicin with particle size of ~3.0 *μ*m. Aerosol characterization showed a FPF and ED of ~40% and >95% when using Qualicaps® capsules. FPF values of engineered microparticles exhibited that HBCD were about 25.48- and 3.01-fold more efficient than a rifampicin alone and lactose formulation [81].

## 4. Dry Powder Coating

Dry powder coating is a potential eco-friendly manufacturing technology to construct particles and powders with desired functionalities. This versatile technology is useful in several industries such as material processing, pharmaceutical sectors, and food industry. Dry powder coating is a one-step, eco-friendly system with several benefits like short processing time, no need of organic solvents, and distinct drying phase. In dry powder coating, fine guest particles fuse to the host particles surface by physical as well as chemical technique. Dry powder coating is categorized into three different categories according to the degree of treatment such as (i) partial coating (discrete coat), (ii) film formation, and (iii) encapsulation (continuous coat) [82, 83]. Various techniques have been utilized for the development of uniform mixtures. Mechanical tumbling, cube mixer, V-blender, and Turbula mixer are commonly used lab scale techniques to prepare uniform mixtures. In contrast, various highly functionalized large scale techniques are also explored and analyzed in the scientific literature (Table 8). Efficiency of dry powder coating is mainly reliant on understanding of powder/particle characteristics specifically particle size, shape, cohesivity, density, surface area, surface energy, surface roughness, and electrostatic charge [82, 83]. Various applications have been investigated in field of dry powder inhalers for improvement of homogeneity, flowability, and dispersibility.

## 5. Force Controlling Agents

In case of DPIs, addition of a tertiary ingredient, force controlling agent (FCA) has indicated potential in enhancing functioning by altering interaction between carrier surface and drug particles. The mechanism of improved aerosolization was assumed to be because of either dissipation of electrostatic charge and capillary forces of ingredients of mixture by FCA or creation of simply dispersible mixed drug-excipient agglomerates when a substantial quantity of a fine excipient as FCA was used [84, 85]. As FCAs, their crucial role is to control interfacial features of carrier particles to reduce drug-carrier adhesion [86]. Simply, a force controlling agent is excipient which lessen the cohesion force between the fine particles within the powder formulation, thus improving deagglomeration upon aerosolization of the powder form DPI device. It is also known as antiadherent or antifriction agent. These excipients may be composed of one or more compounds selected from various categories like amino acids, peptides or a derivative thereof, a metal stearate or a derivative thereof, phospholipids, natural and synthetic lung surfactants, and hydrogenated oils which are solid at room temperature (Table 9).

Amino acids are endogenous molecules which could have less safety issues for delivery to respiratory tract. Amino acid or a derivative thereof having a molecular weight from 0.25 to 1000 kDa is physiologically suitable and acceptable for inhalation delivery. Additionally, D- and DL-forms of amino acids may also be utilized. Amino acids can improve aerodynamic performance of spray-dried powders by reducing moisture absorption and surface tension. Amino acid or a derivative thereof is suitable for delivery of potent biomolecules and bacteriophages [87, 88]. Higher concentrations of essential amino acids can enrich the aerosol pattern of antibiotic DPIs without induction of apparent cytotoxicity [88]. Still, this aerosolization enhancing property seems to be precise for an amphiphilic amino acid, that is, l-leucine, and it is not necessarily the case with other amino acids. Natural and synthetic lung surfactants or water soluble/dispersible surface active ingredients are also used as FCA. When these agents are delivered to the lung, they quickly spread over the large surface of the lung. It is hypothesized that this quick spreading of surfactants might assist in easy dispersion of the therapeutic molecule in formulation, thus helping and improving its pharmacological action. Apart from reducing the cohesion between the fine particles of formulation, additive like metal stearate or a derivative thereof can control the ingress of moisture into the dry powder formulation. Other sugars, for example, glucose, mannitol, maltitol, sorbitol, xylitol, and trehalose, have also been studied as powder coating agents [89].

### 5.1. Amino Acids as Force Controlling Agent

#### 5.1.1. Aerosol Enhancer l-Leucine

l-Leucine (Figure 2) is frequently studied as aerosol enhancer for DPI delivery of numerous drugs for treatment of several respiratory diseases. Leucine has been showed to act as a lubricant for inhalable powders. Aerosolization pattern of DPI can be enhanced by forming an external layer of leucine which is a rather hydrophobic AA with surface activity. Depending on the concentration, leucine frequently heads towards creation of irregular surface morphology that is shown as corrugated or wrinkled surfaces. Such particles would have moderated interparticulate contact points and therefore lesser cohesive or adhesive forces than smooth surface structures. It seems that addition of leucine results in less cohesive particles because of surfactant behavior of leucine [87, 91]. Leucine might also help avoid solid bridge creation between particles. Leucine coating layer may shield amorphous matrix against temperatures and moistures and inhibit crystallization of added excipients in formulation which will enhance inhalable amorphous powder stability. Diverse coating film creation techniques for leucine might influence its purpose; for example, leucine layer formed via solute diffusion was described to give superior protection compared to the case where leucine layer was formed by vapor deposition technique. Leucine was also utilized in mixture with other components [87]. In multicomponent biomacromolecules DPI formulations, leucine worked as a particle structuring and aerosolization enhancing agent [92]. In addition, leucine was also utilized in a Phase I investigation to treat drug-resistant tuberculosis by means of inhaled DPI formulation of capreomycin, without provoking toxicity [88]. Yu et al. studied formulation containing curcumin-chitosan nanoparticle complex (nanoplex) which was produced by spray-freeze drying method. The nanoplex formulation containing leucine/mannitol (0.25%) mixture was dispersible showing satisfactory deposition of curcumin, with FPF of 49 ± 1.0% and ED of >90%, at 60 l/min using Rotahaler inhaler. By way of MTT assay on A549 cell line almost no cytotoxicity of nanoplex was shown. However, it is interesting to note that the nanoplex formulation had much more satisfactory MIC against common pulmonary pathogens than curcumin alone [93]. Chitosan-based leucine conjugated nanoparticle of diltiazem hydrochloride was engineered by customized glutaraldehyde crosslinking method. Aerosol delivery of these engineered nanoparticles using Rotahaler device at a flow rate of 60 l/min resulted in a calculated ED of 79%, a statistically considerable raise in the ED compared to the nonconjugate product (72%). Remarkable improvement in aerosol performance was accredited to amphiphilic surroundings of leucine, hydrophobic environment of crosslinking, and superior swelling properties of nanoparticles [92]. Despite the above stated investigation, in a various study it was revealed that application of leucine as excipient created superior DPI performance. It was also specified that by adding leucine particle size of microparticles reduced while process yield and % FPF improved appreciably. Addition of leucine leads to forming spherical particles with rough surfaces.* In vitro* deposition data revealed that presence of leucine (10%, w/w) resulted in formation of inhalable powders with highest FPF (45%) [94]. Levofloxacin microparticles based DPI formulation was prepared by SD with chitosan and leucine as a model dispersibility enhancer. The developed microparticles showed the better aerosol performance with ED of >90% and FPF of 52.0%. Moreover, microparticles demonstrated better antimicrobial assessment properties without any cytotoxic effect [95]. Leucine has potential of relocation to surface of particle droplet in atomization phase of SD and therefore inhibits water from being cached onto surface producing pitted particles on drying, which have an abridged contact vicinity and accordingly reduced cohesion [95]. Mainly 3 amino acids, glycine, alanine, and leucine, were selected purposely with rising hydrocarbon chain lengths correspondingly, to manage precise features of a spray-dried mannitol based DPI formulation. Glycine or alanine, even if being structurally analogous to leucine, offers unfavorable rather than valuable effects on particles during operation after spray-drying with mannitol. Additionally, blending of leucine with either glycine or alanine (or both) offers particles with potential advantages of leucine. Combined use of three amino acids at 15% was more efficient at improving FPF than combination used at 30%. Highest FPF (68%) was achieved with formulations containing leucine alone [96]. The IgG (immune globulin G) antibody was spray-dried in company of different AA, namely, leucine, phenylalanine, cysteine, glycine, lysine, and arginine, at two different concentrations, that is, 20 and 50% (w/w). IgG containing leucine and arginine at 20% produced the highest and lowest yield (38.2 ± 0.40% and 14.0 ± 0.2%), respectively. The particles ranged in size from 4.17 ± 0.12 mm to 8.11 ± 0.57 mm and the span was between 0.94 and 1.97. Samples containing 50% cysteine and phenylalanine showed significantly higher FPF of 70.51 ± 0.23% and 62.43 ± 0.34%, respectively. While samples containing 50% leucine showed slightly lower FPF (90.6 ± 20.32%) and ED (58.68 ± 0.62). Furthermore, use of cysteine at 50% (w/w) displayed satisfactory stability (2 months at 45°C). These outcomes displayed that phenylalanine and cysteine act as efficient AA for fabrication of IgG DPI regarding stability and aerodynamic behavior [97].

#### 5.1.2. Trileucine

Trileucine is another important AA used in various DPI formulations. It has a high *T*_*g*_ (104°C), which reveals good physical stability of trileucine enclosing respirable powders. *T*_*g*_ of spray-dried trileucine is reliant on pH and can be associated with amount of anion, cation, and zwitterion strength in solution. The solubility of trileucine is based on pH and is lowest at neutral pH (6.8 mg/mL). Trileucine's low aqueous solubility promotes creation of low density corrugated particles and its surface active nature allows formation of powders with reduced cohesiveness. Compared with leucine, trileucine has a superior surface activity and 3-fold lower water solubility and is noncrystalline after SD. Moreover, trileucine competes with protein on air/water edge resulting in an extra depression of surface tension in solution which associates with a reduced denaturation and aggregation in solid state [98].

### 5.2. Metal Stearates as Force Controlling Agent

#### 5.2.1. Magnesium Stearate

Magnesium stearate (MgSt, Figure 2) is also most commonly utilized additive to enhance flow properties of powders; it has been proved to lessen adhesion force owing to its effect on long range van der Waals forces between particles of a powder bed. This is assisted by the fact that hydrophobization of matter, accomplished with MgSt, is identified in adhesion literature to decrease force of adhesion considerably [3]. Favorable MgSt amount, that is, amount which increases powder flow most, can be located when a perfect film has been created to enclose every single particle. Beyond optimal concentration, there is a considerable fall in flowability when the film created is enhanced in thickness or when an exceeding number of fine particles are present [99]. Newly, engineering of lactose carrier surfaces with addition of MgSt has been studied to improve* in vitro *aerodynamic performance of such formulations by reducing adhesion. In these studies, mean of enhanced aerodynamic performance based on particle smoothing was an outcome of superior affinity of MgSt for active sites on exterior of carrier particles that would develop a film to coat dents and thus ease drug dispersion [85]. More recently, feasibility of using new solid coating method named as Mechanofusion® has been investigated to display the potential use of MgSt in optimizing efficiency of carrier based DPIs. Creation of a nanometer-thick coating onto host particles during this highly intensive coprocessing scheme diminishes adhesive interactions between drug and carrier and therefore facilitates the uncoupling of drug particles from carrier upon aerosolization [99]. Various investigations on dry powder coating formulations of inhaled lactose are portrayed in Table 10.

## 6. Miscellaneous Carrier Systems

Hassan and Lau investigated the dispersion and* in vitro* deposition of budesonide loaded pollen-shaped hydroxyapatite loaded particles via Rotahaler at flow rates of 60 l/min. Developed pollen-shaped microparticles showed 85.9 *μ*m particle size and 0.289 g/cm^3^ tapped density. These microparticles were apt for optimum lung deposition with FPF of >40% and ED of >90%. The superior performance of pollen-shaped microparticles was credited to modified surface properties and better powder flowability [105]. Recently, Tuli et al. developed polycaprolactone microspheres loaded SS powders by solvent evaporation technique. Additionally, developed microspheres were coated with dry and wet coating approach using 2% MgSt. Developed spherical microspheres showed rough surface and VMD of 104 ± 0.4 *μ*m. Aerodynamic performance of these dry coated microspheres using Rotahaler at a flow rate of 60 l/min resulted in ED of approximately 50%. Both coating techniques do not show any significant difference in aerodynamic performance. Furthermore, developed microspheres showed 1.17-fold improvement in FPF as compared to *α*-LM (Aeroflo®-95) [106].

## 7. Computational Fluid Dynamics (CFD): Formulation and Device Interaction

Satisfactory drug delivery from DPI device requires adequate fluidization (aerosolization), deagglomeration, and fair dispersion of particles from inhaler device. Effective drug delivery is mainly governed by three factors: (i) interparticulate forces between the powder particles; (ii) deagglomeration and dispersion forces produced throughout inhalation; and (iii) deposition forces in the respiratory tract [3]. Mechanisms of deagglomeration are more complex and involve various constraints like mechanical vibration, influence of turbulence, particle-particle interaction, and mainly the particle-device interaction within the inhaler body [107]. Moreover, the virtual significance of these mechanisms remains uncertain and mainly differs with powder characteristics and inhaler designs (single dose or multidose). By knowing these facts very recently, CFD modeling was utilized dynamically to simulate and investigate deagglomeration/dispersion and airflow mechanisms in DPIs [107].

CFD is a simulation technique in which the dynamic equations governing fluid motion are answered numerically over a physical area of curiosity. These computer-based simulation techniques are very powerful and offer a wide range of industrial and nonindustrial application due to cheaper computing power and easily available commercial software [108]. It has been exercised comprehensively in various disciplines of science (material science) and engineering (electrical, biomedical, and chemical) in the analysis of fluid flows. CFD analysis considers the fluid flow, heat transfer, mass transfer, and newly associated phenomena, for example, marine engineering, environmental engineering (Earth climate systems), hydrology, and oceanography using computer driven simulation. Summary of reports of particle-device interaction is discussed below [108, 109]. CFD approaches are at present increasingly used in the analysis of DPIs, as discussed below.

In 2010, Tong et al. carried out a study to investigate effects of particle size and polydispersity on the dispersion of agglomerates in a cyclonic flow model like the commercial Aerolizer inhaler. CFD-DEM (discrete element method) approach was used to simulate the dispersion of agglomerates to determine particle-device interaction forces. Results proved several particle-device collisions, resulting in a considerable enhancement of agglomerate disintegration. Leading aspects in agglomerate disintegration were recognized as particle-wall impact energy and particle-particle tensile strength. Additionally, PSD was found to have a considerable impact on dispersion pattern; that is, agglomerates with narrower PSD tend to have better dispersion [110]. The same group also undertook a study to examine the dispersion of drug-mannitol agglomerates in tailored impaction geometries using a CFD-DEM model. These simulations showed that deagglomeration was mainly because of particle-wall impactions and fragmentation controlled by agglomerate strength and impaction energy. Results showed that both flow conditions and device design should be judged during analyzing optimal dispersion performance [111]. Furthermore, by using CFD-DEM model same group examined dispersion pattern in the Aerolizer inhaler. CFD-DEM analysis showed that particle-particle collisions were essential only in the initial stages of actuation because deagglomeration was mainly formed due to collisions with the inhaler grid. Simply, in summary, Tong et al. research group offered important insights into dispersion and deagglomeration pattern [112]. Suwandecha et al. utilized a CFD approach to simulate the turbulence kinetic energy (TKE) and particle impaction to study the delivery of particles during the aerosolization processes. In this study, three size ranges of lactose carrier and two devices, that is, Cyclohaler and Inhalator®, are utilized as models for the computational simulations. These simulation results showed that carrier size influenced the probability of deagglomeration at higher flow rate (60 and 90 l/min) but not at lower one (30 l/min). Cyclohaler showed lower TKE and high degree of deagglomeration due to cyclone-like design while Inhalator showed higher TKE due to its narrow geometry and cross grid of the Inhalator was an important factor for deagglomeration of the particles [113].

## 8. Conclusion and Future Perspectives

**Figure 3:**
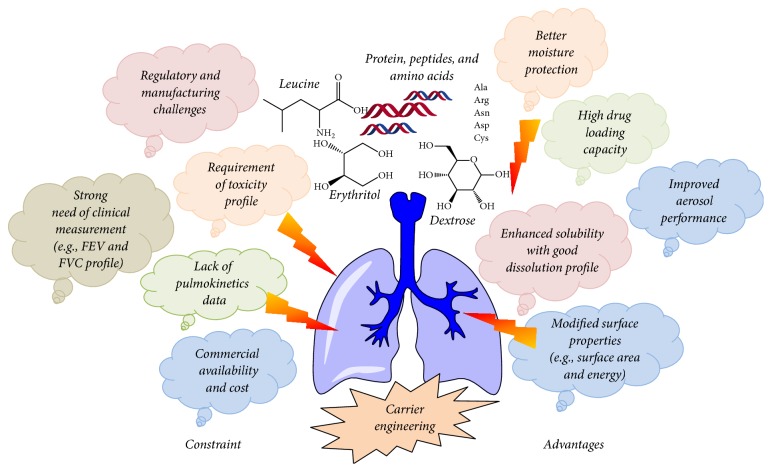
Advantages and constraint in carrier engineering based DPI products.

The existing literature in carrier designing and development for effective pulmonary delivery indicates remarkable progress over the last few years. Carrier modification is a promising tool to defeat various limitations bound to inhaled particles and powders. Superior performance of carrier microparticles depends on few mutually dependent attributes such as carrier shape, size, surface roughness, density, and porosity. Other limits relate to the inhaler device, device metering system, processing conditions, scalability, and reproducibility. Several advantages and constraints in carrier engineering paradigm are given in Figure 3.

Particle engineering provides the alternative of utilizing many carrier's molecules and their combination. Besides traditional ordered or interactive mixing, spray-drying, spray-freeze dying, freeze drying, and supercritical fluid particle development methods can easily be adopted to fabricate inhalable formulations because of the accuracy and easy control over the carrier morphology. Many of the published studies represent the fact that the carrier particle shape and surface morphology can significantly affect the aerodynamic performance of final formulation. An optimum surface roughness is key feature, which improves the aerodynamic dispersion and disposition pattern of a DPI. Among the various particle engineering techniques SD has received better attention for construction of versatile carrier particles due to its relative simplicity, easy scalability, and cost-effectiveness. SD also permits engineering of carrier particles which cannot be easily acquired with other manufacturing techniques. In addition, integration of appropriate stabilizing component during SD might assist in conserving the integrity of biomolecules and enhancing stability of DPI formulations. Also, FD can also be utilized to improve the integrity and stability of numerous therapeutic proteins and peptides by using various cryoprotectants and thermos-protectants (e.g., trehalose dihydrate). Research inputs in this segment led to formation of surface modified sugars and polyols carrier molecules based on dry powder inhaler with better physicochemical stability and enhanced* in vitro* aerodynamic performance.

In addition to particle engineering techniques, dry powder coating is also a capable method to conquer a wide range of DPIs restrictions through enhancement of powder flowability, better dose uniformity, easy deagglomeration during inhalation, and reproducible manufacturing. Mechanism of providing a consistent surface covering on one or more ingredients presents powder with more homogeneous surface area and optimized surface energy for reproducible aerosolization. Furthermore, this method showed useful benefits such as being eco-friendly (no solvents), not necessitating complex multistage processing (one step), being sustainable, being cost-effective, and being easy to scale up. It can be summarized that a number of novel inhalable powders can be manufactured using the wide range of dry particle coating techniques that are presently accessible.

In the last decade, two mannitol based DPI formulations have been acceptable for marketing by US FDA for the treatment and diagnosis of pulmonary disorders. These DPI formulations have given satisfactory outcomes in market. Mannitol based DPI formulations have been permitted for marketing and Aridol®, containing d-mannitol, was permitted by the US FDA in 2010 as a pulmonary diagnostic agent and Bronchitol®, also having mannitol, was permitted for marketing in Australia and Europe in 2011 and 2012, correspondingly, for therapy of cystic fibrosis in adults [44]. Although there are numerous advances in area of carrier designing and development, pulmokinetics, systemic toxicity, and lung clearance rate testing of the engineered carrier are of major importance. To utilize the potential of newly developed carriers in pulmonary delivery, complete attention is needed for pulmokinetics and toxicological problems. Deep understanding of clinical measurements such as FEV and FVC profile of inhaled carriers will also be crucial in assessing their potential for pulmonary delivery. Thus, additional developments focused on alternative carrier to the lung are needed. In terms of carrier engineering, future development remains in the segments of pulmonary pharmacokinetics, pharmacodynamics,* in vivo* deposition efficiency, better stability in pulmonary fluids, systemic toxicity, lung clearance rate, biocompatibility, and most importantly regulatory requirements. At last, this continued advancement will benefit clinicians and patients, allowing easy delivery of various therapeutic agents as dry powder inhalers with improved* in vivo* efficacy, better patient adherence, and reduced adverse effects.

## Figures and Tables

**Table 1:** Hypotheses and working mechanism of “addition of lactose fines” [6, 7].

Hypothesis	Working mechanism
Active sites hypothesis	Fines engage so-called “active sites” (more adhesive sites) on carrier surface, leaving only weaker binding sites (less adhesive sites) accessible for the drug particles to bind to.

Agglomeration hypothesis	Fines materialize agglomerates, multilayers with drug particles, which are hypothetically more easily isolated from the carrier surface.

Buffer hypothesis	Fines coarser than the drug particles might work as a buffer between moving carrier particles and shelter drug particles from press-on forces during mixing.

Fluidization hypothesis	Fines enhance the tensile strength of the powder mixture, which enhances the minimum fluidization velocity (MFV) required for fluidization and therefore the energy available for dispersion.

Case-dependent hypothesis	Contrary to the all above hypothesis, fines do not always enhance the aerodynamic performance of a DPI which is concluded by the formulation and dispersion situations.

**Table 2:** Types of lactose used for DPI formulation and their suppliers.

Region	Lactose suppliers with brands
America	Sheffield Pharma Ingredients (Monohydrate Inhalation®)
	Foremost Farms USA

Europe	Meggle Pharma GmbH (InhaLac®)
	^*∗*^DMV-Fonterra Excipients (Respitose)
	^*∗*^Friesland Foods Domo (Lactohale®)

Australia and Newland	^*∗*^Lactose New Zealand (Wynhale®)

^*∗*^Lactose New Zealand, Friesland Foods Domo, and DMV-Fonterra Excipients, joint venture.

**Table 3:** Excipients suitable for use in DPIs-based pulmonary delivery.

Excipients	Molecular formula	Molecular weight (g/mol)	^#^Lethal dose (g/kg)
*Sugar carriers*			
*α*-Lactose monohydrate	C_12_H_24_O_12_	360.310	>10^*∗*^
Mannitol	C_6_H_14_O_6_	182.172	13.5
Trehalose dihydrate	C_12_H_26_O_13_	378.327	5
Sorbitol	C_6_H_14_O_6_	182.170	17.8^*∗∗*^
Raffinose pentahydrate	C_18_H_42_O_21_	594.513	-
Maltose monohydrate	C_12_H_24_O_12_	360.312	34.8
Dextrose monohydrate	C_16_H_14_O_7_	198.171	25.8
Xylitol	C_5_H_12_O_5_	152.146	17.3
Erythritol	C_4_H_10_O_4_	122.120	13
Sucrose	C_12_H_22_O_11_	342.297	29.7
*Cyclodextrins*			
*α*-Cyclodextrin	C_36_H_60_O_30_	972.846	0.79^*∗∗∗*^
*β*-Cyclodextrin	C_42_H_70_O_35_	1134.987	18.8
*ϒ*-Cyclodextrin	C_48_H_80_O_40_	1297.128	8.0
*Metal stearate*			
Magnesium stearate	C_36_H_70_O_4_Mg	591.257	>10^*∗*^
Calcium stearate	C_36_H_70_O_4_Ca	607.030	>10 and >1241^##^
Zinc stearate	C_36_H_70_O_4_Zn	632.332	>10 and >1241^##^
*Amino acid*			
Leucine	C_6_H_13_NO_2_	131.175	-
Trileucine	C_18_H_35_N_3_O_4_	357.495	-

^#^Lethal dose (LD50): in rats after oral administration. ^*∗*^IIG Limits as per USFDA-*α*-LM: 24.95 mg and Mg. Stearate: 0.13 mg. ^*∗∗*^Lethal dose (LD50): in mouse after oral administration. ^*∗∗∗*^Lethal dose (LD50): in rat after intravenous administration. ^##^Lethal concentration (LC50): in mammal after inhalation.

**Table 4:** Crystallized lactose based DPI formulations.

Drug	Method	Key ingredients	Device and flow rate	% FPF	References
			(l/min)		
SS	ASC	Lactochem and acetone : water (85 : 15)	Rotahaler and 60	26.9 ± 1.8	[23]
SS	ASC	Ethanol : butanol (20 : 60)	-	38.0 ± 2.5	[24]
SS	ASC	Ethanol : acetone (40 : 40)	Aerolizer and 92	40.48 ± 4.57	[25]
SS	ASC	Pharmatose 100 M and ethanol : butanol	Aerolizer and 92	-	[8]
SS	ASC	Pharmatose 100 M and butanol : acetone (80 : 00)	Aerolizer and 92	-	[26]

ASC: antisolvent crystallization; SS: salbutamol sulphate.

**Table 5:** Spray-dried lactose loaded DPI formulations.

Drug	Method	Key	PSD	Device and flow	% FPF	References
		ingredients	(*μ*m)	rate (l/min)		
Pranlukast hydrate	SD	Pharmatose 200 M and Pharmatose 325 M	<90	Spinhaler® and 60	28.5 ± 3.1	[35]
Human serum albumin	SD	Albumin : lactose : DPPC (30 : 10 : 60)	6.50	Spinhaler and 28.3	41 ± 5.0	[36]
SS	SD	Lactochem crystals	77.72	Cyclohaler and 60	31.3 ± 1.3	[37]
Budesonide	SD	*α*-LM	>230	^*∗*^Taifun®	31.0 ± 5.1	[38]

^*∗*^Taifun is a multiple dose, reservoir-based DPI. DPPC: dipalmitoyl phosphatidylcholine; FPF: fine particle fraction.

**Table 6:** Crystallized mannitol based DPI formulations.

Drug	Method	Key ingredients	PSD (*μ*m)	Device and flow rate (l/min)	% FPF	References
SS	ASC	Mannitol and acetone : water (95 : 5)	94.49 ± 8.92	Aerolizer and 92	43.99 ± 2.62	[45]

SS	ASC	Mannitol and ethanol : water (90 : 10)	148.57 ± 3.51	Aerolizer and 92	45.8 ± 0.7	[46]

SS	ASC	Mannitol and ethanol : acetone (40 : 40)	-	Aerolizer and 92	38.6 ± 2.9	[47]

SS	CC	Mannitol 20%, w/v, and deionized water	-	Aerolizer and 92	33.8 ± 1.2	[20]

SS	ASC	Mannitol : Pharmatose 100 M (10 : 10) and acetone : water (80 : 20)	73.8 ± 0.6	Aerolizer and 92	38.6 ± 3.2	[50]

SS: salbutamol sulphate; ASC: antisolvent crystallization; CC: cooling crystallization; and FPF: fine particle fraction.

**Table 7:** Microcomposites carrier loaded DPI formulations.

Drug	Method	Key ingredients	PSD (*μ*m)	Device and flow rate (l/min)	% FPF	References
Rifampicin	Four-fluid nozzle SD	PLGA and CM	4.50	Jethaler and 28.3	~35–40	[58]
Ciprofloxacin HCL	co-SD	CM	3.4 ± 0.1	Cyclohaler and 60	43.5 ± 1.5	[59]
Meloxicam	co-SD	CM, PVA, and leucine	5.81	RS01® and 60	53.53 ± 2.02	[60]
Theophylline	Wet bead milling followed by SD	CM	5.11 ± 1.22	Cyclohaler and 60	56.8 ± 8.7	[61]

SD: spray drying; co-SD: co-spray drying; CM: commercial mannitol; PVA: polyvinyl alcohol; PLGA: poly(lactic-co-glycolic acid); and FPF: fine particle fraction.

**Table 8:** Dry powder coating technologies for manufacturing versatile powders/particles [82, 83, 90].

Technologies	Speed/shear rate	Functionality/principle	Limitations
Cyclomix	Rotor speed up to 30 m/s and high shear	Mild coating technique. Specifically, designed for cohesive powders with liquids or melt binders and with excellent temperature control system.	Not applicable for fragile host material. May lead to particle attrition or fracture of host materials.

Fluid energy mill	Micronization coating	Most commonly used for fine grinding and close particle size control (less than 10 *μ*m).	Less mechanical force produced compared to other high force technologies. Continuous attrition of particles generating amorphous form or different polymorphs. Need to monitor various variables to ensure desired particles morphology.

Hybridiser	5000–20,000 rpm and high shear with compression forces	Temperature based fusion with ensuing good homogeneity. Producing satisfactory physical and/or chemical bond within very short procession time.	Not suitable for thermolabile and fragile material and may lead to particle attrition or fracture of host materials. May generate amorphous form or change chemical and electronic status of material. Not suitable for continues process.

Mechanofusion	200–10,000 rpm and high shear	Temperature based fusion between guest and host particles. Generating very strong physical and/or chemical bonding.	Not applicable for thermolabile and fragile host material. Not suitable for continuous process.

Magnetically assisted impaction coating	High shear force and use of magnetic field	Soft coating technique suitable for thermolabile material. Minimum impact on particle shape and size.	Not suitable for continuous batch process. High risk of contamination. Less mechanical force produced compared to other high force technologies.

Rotating fluidized bed coater	Centrifugal fluidization and shear forces	Soft coating technique suitable for smaller size small host and guest particles. Suitable for continuous batch process operation.	Unnecessary aerosolization of fine guest particles. Less mechanical force produced compared to other high force technologies.

Theta-composer (elliptical rotor)	500–3000 rpm and shear stress with compaction force	Soft coating and no thermal deterioration with short processing time of 2–10 min. No major change in particle size or shape.	Not suitable for continuous batch process. Less mechanical force produced compared to other high force technologies.

Turbo rapid variable	Impeller tip speed up to 20 m/s	High shear blending unit contains a single, bottom driven impeller drive. Suitable for continuous batch process operation and provide homogenous product.	Not applicable for thermolabile and fragile host material.

**Table 9:** Force controlling agents commonly used in dry powder inhalers [89].

Category	Force controlling agents
Amino acids (AA) and derivatives	L-Leucine, isoleucine, trileucine, lysine, methionine, phenylalanine, valine, aspartame, and acesulfame potassium

Natural and synthetic lung surfactants lipids and phospholipids	Dipalmitoyl phosphatidylcholine (DPPC), dipalmitoyl phosphatidylethanolamine (DPPE), dipalmitoyl phosphatidylinositol (DPPI), phosphatidylglycerol (PG), lecithin, soya lecithin, laxiric acid and its salts (sodium lauryl sulphate and magnesium lauryl sulphate), triglycerides (Dynasan® 118 [a microcrystalline triglyceride], Cutina® HR [hydrogenated castor oil waxy powder])

Saturated fatty acid and derivatives	Behenic acid, erucic acid, lauric acid, oleic acid, palmitic acid, stearic acid, and glyceryl behenate

Natural and synthetic minerals	Aluminum dioxide, silicon dioxide, starch, talc, and titanium dioxide

Metal stearates and derivatives	Calcium stearate, lithium stearate, magnesium stearate, sodium stearate, zinc stearate, sodium stearoyl lactylate, and sodium stearyl fumarate

**Table 10:** Dry powder coated lactose incorporated DPI formulations.

Drug	Coating mechanism and coating time (min)	Key ingredients	PSD (*μ*m)	Device and flow rate (l/min)	% FPF	References
SS	Wurster Fluidized Bed and 180	Pharmatose 200 M and aqueous HPMC solution	81.80	Jethaler and 60	34.9 ± 3.7	[100]
SS	Theta-Composer® and 10	Pharmatose 200 M and MgSt (10%)	71.8 ± 1.75	Jethaler and 30	~40.00	[99]
SS	Vortex-Mixer and 60	Pharmatose 200 M and MgSt (2%)	87.50	Jethaler and 60	26.0 ± 3.9	[101]
SS	Theta-Composer and 10	Pharmatose 200 M and sucrose tristearate (10%)	71.9 ± 17.5	Jethaler and 60	46.86 ± 1.5	[102]
TNRA	Rotor-type powder mixer (Mechanofusion) and 15	Pharmatose 325 M and sucrose stearate (1%)	90	Jethaler and 30	20.90	[103]
TNRA	Rotor-type powder mixer (Mechanofusion) and 15	Pharmatose 325 M and MgSt (2%)	85.0 ± 2.0	Jethaler and 30	34.60	[103]
SS	Rotor-type powder mixer (Mechanofusion)	MgSt (5%)	-	Monodose inhaler and 60	69.00	[104]
Salmeterol xinafoate	Rotor-type powder mixer (Mechanofusion)	MgSt (5%)	-	Monodose inhaler and 60	73.10	[104]

SS: salbutamol sulphate; MgSt: magnesium stearate; TNRA: triple neurokinin receptor antagonist; and FPF: fine particle fraction.
